# Understanding the impact of unequal utilization of medical services on the consumption of resources in hemophilia

**DOI:** 10.1186/s12962-022-00409-5

**Published:** 2022-12-30

**Authors:** Lele Li, Xiaotong He, Yixuan Li, Xiangyu Chen, Xue Wang, Chunhou Li

**Affiliations:** 1 School of Labor and Human Resources, Renmin University of China, Beijing, 100872 China; 2 Peking Union Medical College Hospital, Chinese Academy of Medical Science, Beijing, 100730 China

**Keywords:** Utilization of medical services, Consumption of medical resources, Hemophilia patients, Accessibility of medical services, Inequity

## Abstract

**Objective:**

Unequal utilization of medical services will have a significant impact on the consumption of resources entitled through health insurance claims of patients. With the proposal of the goal of Healthy China, it is essential to achieve the equity of medical service utilization of patients with rare diseases to further promote social equity and justice. To analyze the consumption of medical resources of rare disease patients, so as to explore how different medical insurance types impact patients’ medical resources consumption. This study pioneeringly used medical data from Peking Union Medical College Hospital. By analyzing the consumption data, this paper fills the research gap of existing studies on the analysis of medical service utilization inequality based on the perspective of insurance participation by examining the effects of different types of insurance coverage on medical resource consumption of hemophiliacs. At the same time, rare disease patients as a minority group have long been neglected in the analysis of medical resource consumption, and this paper fills the research gap of medical service utilization of this group to a certain extent by taking hemophiliacs as the object of analysis.

**Methods:**

Based on the medical data of hemophilia patients in Peking Union Medical College Hospital from 2010 to 2020, we analyze the consumption of medical resources of the patients, and use a multiple regression model to explore how different medical insurance types impact patients’ medical resources consumption.

**Results:**

The study has three main findings. Firstly, the disparity in medical expenditure among different hemophilia patients is quite obvious, and the drug expense accounts for a large proportion of the total cost. Secondly, the ratio of reimbursement is generally low, and there is a wide difference in the amount of reimbursement for different types of medical insurance. Thirdly, the resource consumption of patients with different medical insurance types differs a lot.

**Conclusion:**

In order to improve the current medical insurance system and mechanism for providing orphan drugs, we put forward the following suggestions. First of all, the medical security system should expand its coverage to eliminate the medical expenditure gap between different hemophilia patients, therefore better promoting equity under the same insurance type. At the same time, the reimbursement level of medical expenses for patients with rare diseases should be appropriately improved, so as to reduce the financial burden of patients with rare diseases, and promote the realization of economic equity. In addition, society should pay special attention to the disadvantaged groups, reduce the differences between various medical insurance, and improve the level of health and equity of the whole society.

## Introduction

Hemophilia is a kind of disease caused by the lack of coagulation due to gene mutation [1], and gene therapy is the only radical cure for it. In view of safety, conventional drugs are normally used to treat hemophiliacs. As hemophilia cannot be cured by normal medicine, the high cost and long period of treatment usually make the medical expenditure very high [2]. In recent years, rare diseases have become a growing concern. In 2018, China has released its first directory on rare diseases, including 121 kinds of rare diseases. In 2020, the State Council proposed to explore the mechanism of providing orphan drugs and strengthen the top-level design of rare disease security system.

Peking Union Medical College Hospital is one of the top hospitals in China for the treatment of hemophilia. In Beijing, there are only three hospitals that are equipped to accommodate hemophilia patients, with pediatric patients generally being treated at the Beijing Children’s Hospital and adult patients being treated at the Union Medical College Hospital and the Peking University People’s Hospital. The number of hemophiliacs admitted to the Union Hospital accounts for 50–60% of all patients in Beijing. The present study, however, is based on the full sample of patient data provided by the Peking Union Medical College Hospital over the years, which has the advantages of large sample size and wide sample coverage over time, and thus the results of this paper can be considered to be of some reference significance in interpreting the consumption of medical resources by hemophiliacs in China.

China’s 14th five-year plan has clearly put forward the goal to promote the construction of Healthy China, and improve the overall medical insurance system. The social medical insurance system plays an important role in improving the health level of the whole society and promoting social equity and justice. It is especially important to ensure the equity of medical services utilization of patients with rare diseases. Therefore, we use the medical resources consumption data of hemophilia patients in Peking Union Medical College Hospital from 2010 to 2020, construct a theoretical framework to analyze inequity of medical services utilization, and focus on its impact on medical resources consumption. Through our study, we hope to provide empirical evidence for building a more equal and reasonable medical insurance system and improving the orphan drug policies of China.

Equal utilization of medical services is of great significance to achieve social equity and justice.

Rawls put forward two principles of justice in his theory. The principle of equality requires opportunities are equal and open to all people in society while allowing a certain degree of inequality between individuals. Positive measures should be taken to improve the condition of the least-advantaged members [3]. Daniels extended Rawls’ theory to the field of health and medical services. He proposed that Rawls’ principles can only be achieved under the condition that people are able to fully perform their physiological functions during their life cycle and avoid the risks of disease, disability or premature death [4]. Therefore, medical services contribute to the realization of equal opportunity by ensuring people’s capability to participate in social, political and economic life through the prevention and treatment of diseases and disabilities [5]. On the basis of Rawls’ principles, Roemer attributed individual advantage to environment and effort. Society should compensate for the disadvantages caused by environment and encourage the advantages caused by individual effort [6]. Ma et al. linked the compensation and encouragement rules of Roemer to the concept of equity in health economics. He proposed that the compensation rule accommodates horizontal equity and encourage rule accommodates the vertical equity [7]. Therefore, the goal to achieve equal opportunity in medical services is not to provide homogeneous services for all people but to eliminate unreasonable inequity caused by registered residence, institutional settings and so on [8].

Currently, studies on the utilization of medical services mainly relied on the perspectives of family or individual. In 1968, Anderson established the Behavioral Model of Health Services Use model to analyze the impact of tendency characteristics, promotion resources and needs on the utilization of family health services [9]. The Health Human Capital model mainly analyzes the determinants of medical service utilization from the perspective of individual characteristics. Grossman regarded health as a kind of capital stock, and explained the relationship between demographic variables such as age, gender and income level and the demand for medical and health services [10]. Afterwards, Dardanoni & Wagstaff investigated the impact of uncertainty on medical resource consumption. Both the uncertainty of disease incidence and the effectiveness of medical care services will affect patients’ consumption of medical resources [11].

Based on the theoretical model, a large number of empirical studies have shown that under the coverage of different medical insurance systems, there are significant differences in the utilization of medical services among people with different demographic and socio-economic characteristics. It can be concluded into three aspects as follows.

Firstly, people covered by different types of medical insurance systems will have unequal access to medical resources.

China’s basic medical insurance system is mainly composed of Urban Employees’ Basic Medical Insurance (UEBMI), Urban Resident Basic Medical Insurance (URBMI) and New Cooperative Medical System (NCMS). The differences in the security level and scope make different groups have different access to medical resources, resulting in the disparity in consumption of medical resources. Related studies have shown that UEBMI better covers the daily diagnosis and treatment needs of insured patients, so the hospitalization rate and outpatient rate of patients with UEBMI are relatively high [12]. At the same time, it also increases unnecessary medical behavior to some certain extent, resulting in a waste of medical resources. Taking patients with chronic diseases for example, the irrational medical-seeking behavior of patients covered by UEBMI is significantly higher than that of other types of insured patients [13]. NCMS has significantly improved the medical treatment rate of people living in the countryside [14]. But most of the insured patients usually go to community hospitals and private institutions [15], so the insufficient guarantee level of NCMS has directly affected the rural residents, resulting in the high demand of hospitalization [16]. In addition to the basic medical insurance system, commercial insurance can also significantly change the medical behavior of patients [17]. Patients which have both basic medical insurance and commercial insurance enjoy a relatively high level of protection, and they will be more likely to receive treatment when needed. However, when it comes to the diseases that cannot be covered by neither commercial insurance nor basic medical insurance, the average medical expenses of patients are much higher, resulting in unreasonable medical expenditure [18]. In addition to these three types of national health insurance, there are also other supplementary health insurance and commercial insurance. In the regression model below, we classify the participation of the sample into six categories according to the three types of national health insurance that hemophiliacs have and the participation of other commercial insurance.

Secondly, people with different demographic characteristics will face different risks of diseases, so the utilization of medical services will be affected by age, gender and other factors.

Empirical research shows that individual health condition displays a downward trend during the whole life cycle [19, 20]. The decline of health condition indicates the depreciation rate of health capital increases with age. Age is one of the important factors leading to the rise of medical service consumption in the life cycle. As to gender, the relevant data shows that during the life cycle, the medical expenditure of women aged 20–40 is significantly higher than that of men of the same age. But the gender difference will become less obvious after the age of 60 [21]. It means that, compared with men, women have higher risk of diseases during their golden age of childbirth, which will further lead to the difference in medical resources consumption.

Thirdly, the utilization of medical services is closely associated with the socio-economic characteristics of the family, therefore health inequity is often intertwined with poverty.

Studies show that the inequality of medical services expenditure is related to the income level to some extent, and it becomes more evident as age grows [22]. Inequality of opportunity in the health status of the population is closely related to its family and social background, which means that the socioeconomic status and health level of parents will affect future generations through intergenerational transmission [23]. Individual health condition can also be affected by their living areas [24]. The study on European inequality of opportunities in health has found that inequality of opportunities is caused by social reproduction in most countries, and the different extent of inequality between countries indicates the importance of institutional arrangements in promoting social equity[25].

To sum up, scholars have made rich research on the equity of medical service utilization, which laid a solid theoretical and empirical foundation for our study. However, it is worth noticing that the existing studies still have some limitations. Firstly, when analyzing the phenomenon of unequal medical service utilization, most studies mainly focus on the differences between urban and rural areas, or differences caused by age or income gap, few studies comprehensively analyze the differences brought by different types of medical insurance. Secondly, there are rare empirical analysis on the consumption of medical resources by patients with specific diseases, especially lacking attention to patients with rare diseases. Therefore, starting from existing problems, this paper will focus on patients with hemophilia, and comprehensively investigate the impact of different types of medical insurance on the medical resource consumption of patients.

This paper explores the impact of different types of health insurance on the consumption of medical resources for patients with rare diseases in China based on the medical cost settlement data of hemophiliacs with different health insurance participation status from 2010 to 2020 provided by Concord Hospital. By setting each insurance type as a dummy variable into the regression model while controlling for basic patient data such as age and gender, in order to discuss the effect of heterogeneity in participation status on medical costs of hemophilia patients, and then explore the impact of participation on the unequal distribution of medical resources for patients with rare diseases represented by hemophilia and the mechanism of action. The empirical regressions and graphical plots in this paper are based on R language.

The possible marginal contributions of this paper are as follows: First, the existing studies on the causes of inequality in healthcare service utilization mainly focu**s** on the differences in individual characteristics such as urban and rural areas, age, and income, and discuss the heterogeneity of these environmental factors on patients’ healthcare service utilization, while this paper analyzes the impact of different insurance coverage on healthcare service utilization of hemophilia patients from the perspective of insurance coverage, which complements the research on healthcare service utilization caused by insurance coverage. The disparity in reimbursement rates and the resulting differences in patients’ out-of-pocket payments are one of the major causes of the unequal utilization of health care services among patients with major diseases in China. Second, most of the analyses on medical service utilization and medical resource consumption are focused on chronic diseases and common diseases, while rare diseases, as a small “minority” group, have received little attention, butthe medical costs of these patients are often high and the resulting inequality in medical resource consumption is even more dangerous and deserves to be analyzed. This is worth analyzing. In conclusion, this paper investigates the inequality in health care resource consumption among hemophiliacs due to insurance coverage, which complements the gaps in the two aforementioned literatures and has both theoretical and practical implications. Methods.

## Methods

The impact of different types of insurance on the consumption of medical resources is related to the specific design of the insurance system, which varies widely within the Chinese social health insurance system, with barriers between different insurance systems and different levels of coverage, resulting in different out-of-pocket ratios for different types of insured people suffering from the same disease. The inherent differences in China’s health insurance system have a direct impact on the distribution of actual health care resources among different cohorts of insured persons, which in turn has a direct impact on access behavior and the equal distribution of health care resources.

**Fig. 1:**
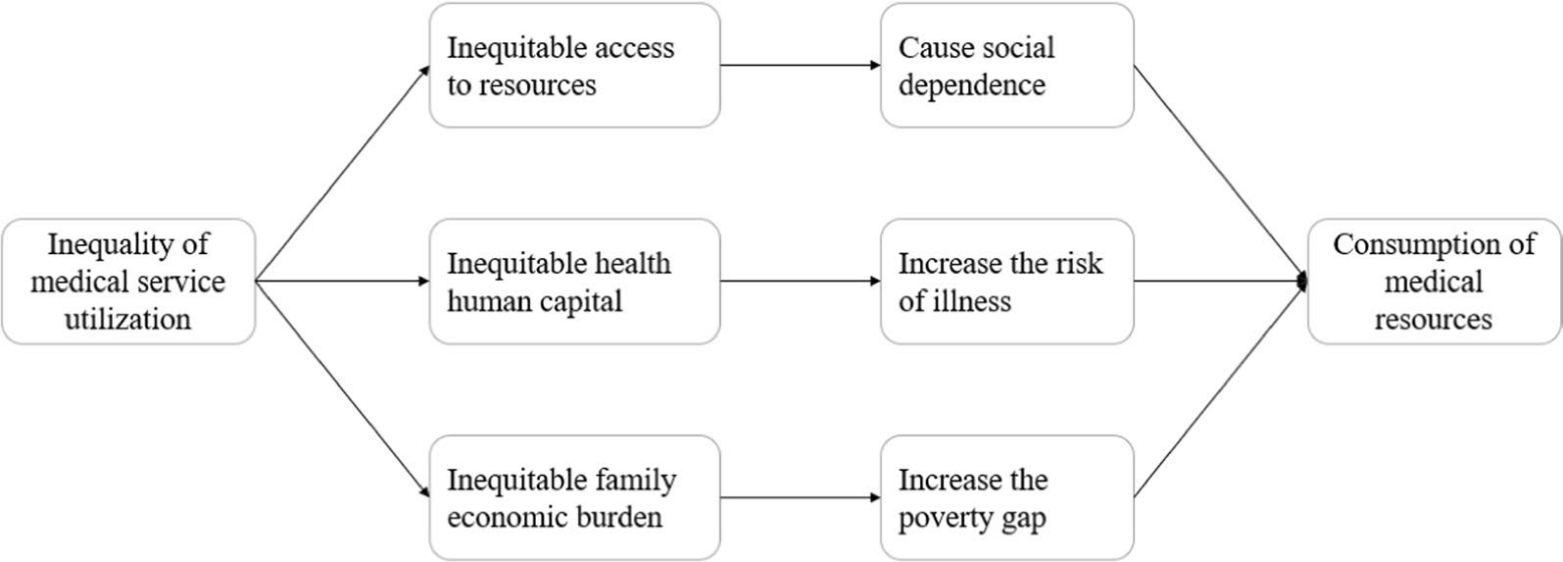
Theoretical framework of medical services utilization and medical resources consumption of hemophilia patients

Combined with relevant literature analysis, we put forward the theoretical analysis framework of medical service utilization and medical resource consumption of hemophilia patients (Fig. 1). Different types of medical insurance are important factors leading to the unfair use of medical services. The unfair use of medical services for hemophilia patients is embodied in the following three aspects. First, patients with different types of medical insurance face different accessibility to medical resources, and this inequality will cause social dependence of some groups, therefore affecting the consumption of medical resources. Second, patients with different individual characteristics have different health human capital due to the unfair use of medical services. Therefore, patients of different ages and gender face different disease risks, resulting in different consumption of medical resources. Third, the unequal use of medical services will also lead different financial burdens for different families. Long-term hemophilia medication and high treatment costs will increase the poverty gap between patients and families, which has a further impact on the consumption of medical resources.

### Data sources and variable settings

The data in this paper are derived from the transaction information of hemophilia patients in Peking Union Medical College Hospital from 2010 to 2020. The data include the basic information of patients, such as insurance type, disease diagnosis information, gender, age, length of stay, and medical expenses, such as sub-total expenses, sub-item expenses, personal payment expenses, and overall fund payment expenses. Peking Union Medical College Hospital is the national leading hospital of China rare disease diagnosis and treatment guidance center and national rare disease diagnosis and treatment cooperation network, and its data is more representative. After eliminating relevant missing values and samples that do not meet the research requirements and controlling all variables, the total number of diagnostic record data used in this paper is 22,837.

In this paper, we focus on the effect of the type of health insurance on the health care resource utilization of patients with hemophilia; therefore, referring to the established literature [26, 27] this paper measures the effect of different types of insurance participation on the health care resource consumption of patients by setting dummy variables to distinguish patients’ participation. In this paper, the explanatory variable is the type of medical insurance that patients participate in, corresponding to the type of insurance in patient information, according to the current medical insurance system in China is divided into six categories:the first category is the basic medical insurance for urban employees; the second category is basic medical insurance for urban and rural residents; the third category is supplemental healthcare insurance, this sample mainly refers to the student and child serious illness medical insurance ; the fourth category is commercial insurance, in this sample for the type of insurance corresponding to unemployed residents of patients ; the fifth category is patients with two or more types of insurance in the above four types ; the sixth category refers to the persons who are unsupported and orphaned or whose legal working age has been determined by the relevant departments to be completely disabled and unable to enter the social insurance system. Therefore, five dummy variables are used in the model to represent the patient’s insurance participation status.

**Fig. 2:**
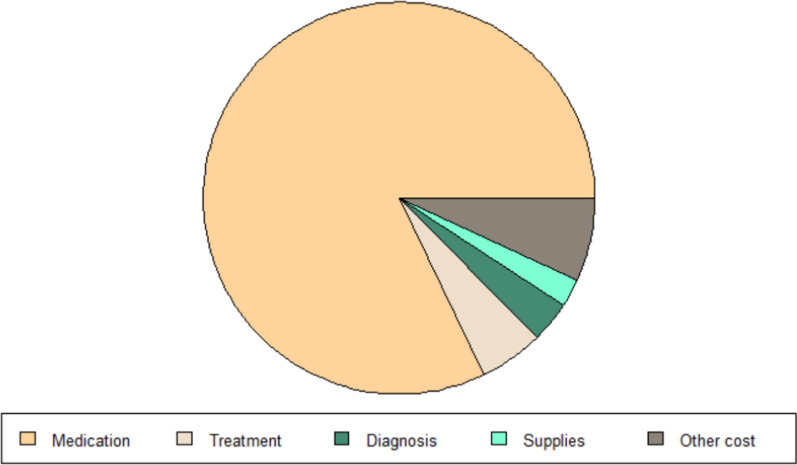
Composition of outpatient and emergency costs

**Fig. 3:**
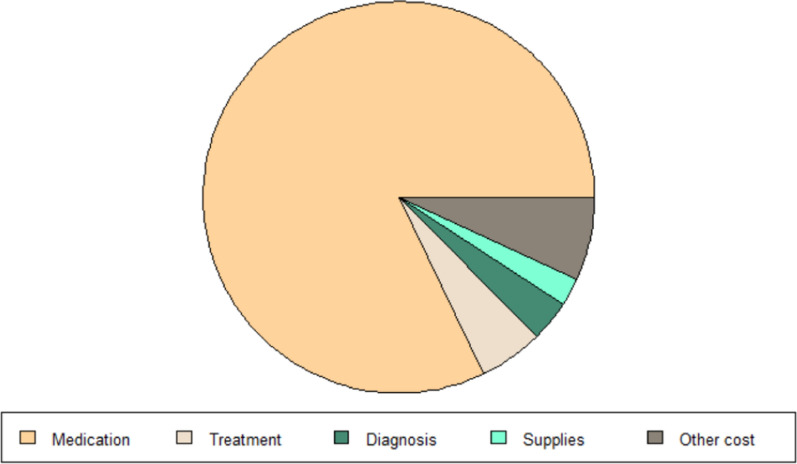
Composition of outpatient special disease

The explained variable in this paper is the consumption of medical resources of hemophilia patients, and medical expenses are the direct embodiment of their consumption of medical resources. Therefore, this paper uses the final medical expenses of hemophilia patients to measure. Since the diagnosis and treatment of hemophilia patients are more than that of outpatient and emergency departments, this paper mainly discusses the outpatient and emergency expenses and outpatient and special expenses of hemophilia. Figures 2 and 3 show the total medical expenditure and each sub-item expenditure of hemophilia patients from 2010 to 2020. Table 1 is a descriptive statistics of patient medical costs and sub-item expenditure. Regardless of the type of medical insurance coverage of patients, drug costs are the main part of medical costs, accounting for more than 80% of total medical costs.

**Table 1:** Descriptive statistics of medical expenses variables

Variables	Outpatient and emergency costs	Mean	Standard deviation	Outpatient special disease	Mean	Standard deviation
Total cost	4341	965.3	2019.0	18.401	6338.9	8603.3
Medication cost	4341	810.4	1993.0	18.401	6330.4	8765.7
Treatment cost	4341	53.1	247.5	18.401	27.9	183.8
Supplies cost	4341	22.8	247.8	18.401	35.8	175.7
Diagnosis cost	4341	33.1	497.3	18.401	14.0	102.5

In terms of control variables, gender, age and disease diagnosis types are controlled in turn. Specifically, the gender variable is a two-class variable, the male assignment is 1, and the female assignment is 0. Since the genetic type of hemophilia is X-linked recessive inheritance, its incidence in men is significantly higher than that in women, so there are more male patients in this sample. Age is a continuous variable, the average age is 39.05 years old, indicating that the sample is relatively young. Different types of disease diagnosis mean different complications and comorbidities for patients, thus affecting the consumption of medical resources by patients. Since it is not a key variable in this paper, we only represent it by the number of complications.

#### Empirical model

We use the data of patients to investigate whether the utilization level of medical services will affect the consumption of medical resources of patients. The empirical model is as follows:

$${EX}_{i}= {\beta }_{0}+{\beta }_{1}{MEDICTYPE}_{i}+{\beta }_{2}{AGE}_{i}+{\beta }_{3}{GENDER}_{i}+{\beta }_{4}{TYPE}_{i1}+{\beta }_{5}{TYPE}_{i2}{+\beta }_{6}{TYPE}_{i3}+{\beta }_{7}{TYPE}_{i4}+{\beta }_{8}{TYPE}_{i5}+\gamma {X}_{i}+\epsilon$$

Among them, ${EX}_{i}$ is the medical cost of patient i. ${AGE}_{i}$ is the age of patient i, ${GENDER}_{i}$ is the gender of patient i, and ${MEDICTYPE}_{i}$ is the disease type of patient. ${X}_{i}$ is the other factors that need to be controlled affecting medical expenses, including patient-level characteristic variables (diagnosis and treatment date, diagnosis and treatment willingness) and hospital-level characteristic variables (different diagnostic doctors, different diagnostic techniques). ${TYPE}_{i}$ is the type of medical insurance for patient i and a key variable of the model. ${TYPE}_{i1}$、${TYPE}_{i2}$、${TYPE}_{i3}$、${TYPE}_{i4}$、${TYPE}_{i5}$ represents basic medical insurance for urban employees, basic medical insurance for urban and rural residents, commercial insurance, supplementary medical insurance, and mixed insurance, respectively. They are virtual variables, when the variable is 1 for this insurance, not 0 for this type of insurance. When all of them are 0, it means that the patient has no medical insurance.

Based on the above theoretical analysis, ${\beta }_{1}$ measures the impact of the number of diseases on patients ' medical resources consumption, the coefficient should be positive, indicating that the more the number of diseases, the more patients consume medical resources. We think ${\beta }_{2}$ should be positive, indicating that the consumption of medical resources in hemophilia patients increases with age. The symbol for measuring the impact of gender on the consumption of medical resources is difficult to predict, as the relationship between gender and the severity of the disease and the treatment of the disease is unclear and it is difficult to determine the direction of its impact in advance. ${\beta }_{4}$、${\beta }_{5}$、${\beta }_{6}$、${\beta }_{7}$、${\beta }_{8}$ separately measures the medical resource consumption of patients with the type of health insurance they represent relative to patients without medical insurance.

## Results

### Medical resource consumption and reimbursement of patients

**Fig. 4:**
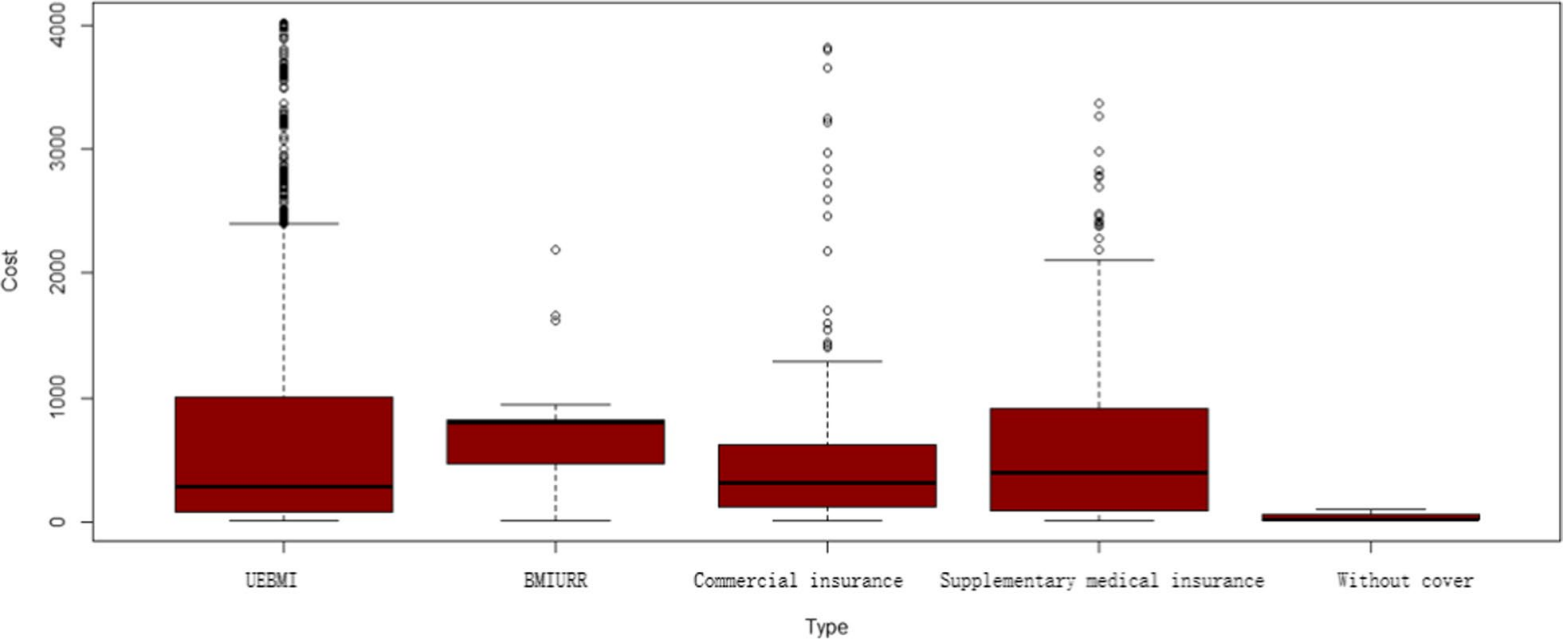
Outpatient expense

**Fig. 5:**
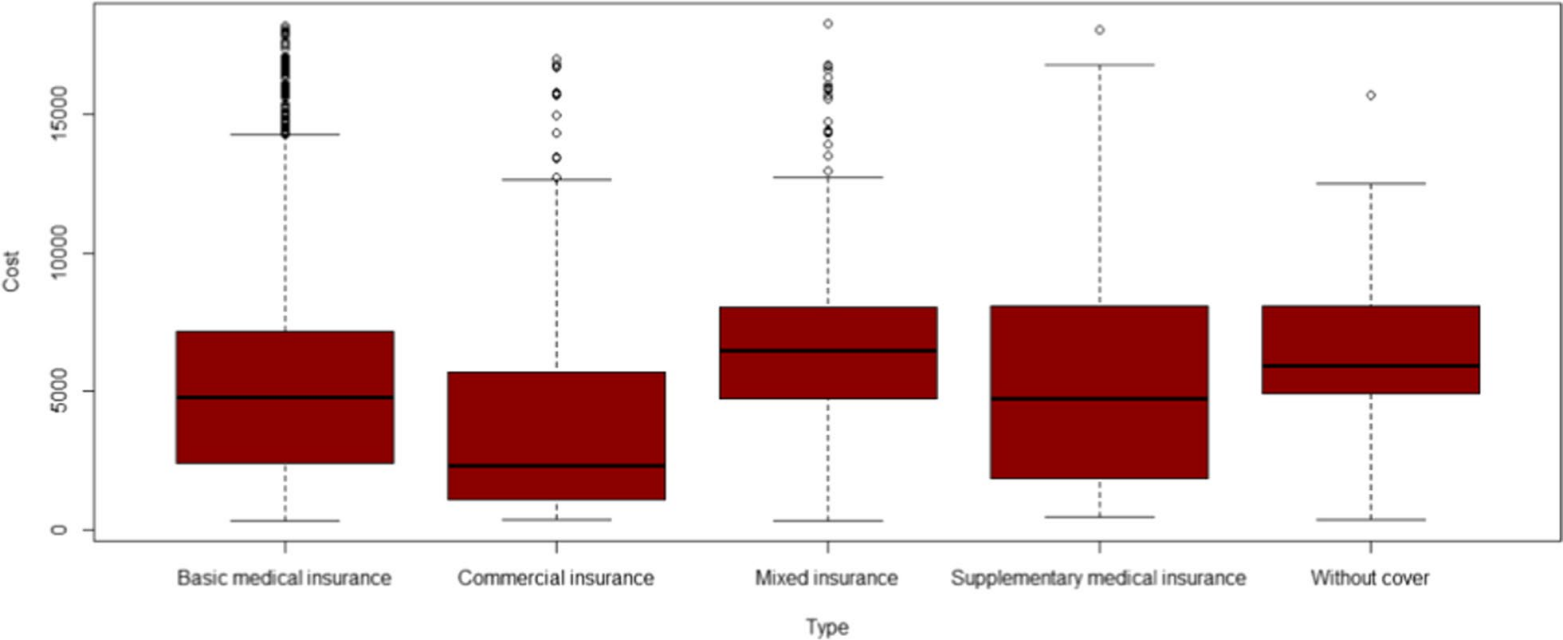
Outpatient special disease expenses

Figures 4 and 5 report the medical expenditure of patients with hemophilia. In terms of medical expenses, the expenses of different hemophilia patients are quite different. In the outpatient special disease expenses, there are a few patients’ medical expenses up to 100,000 yuan.

**Fig. 6:**
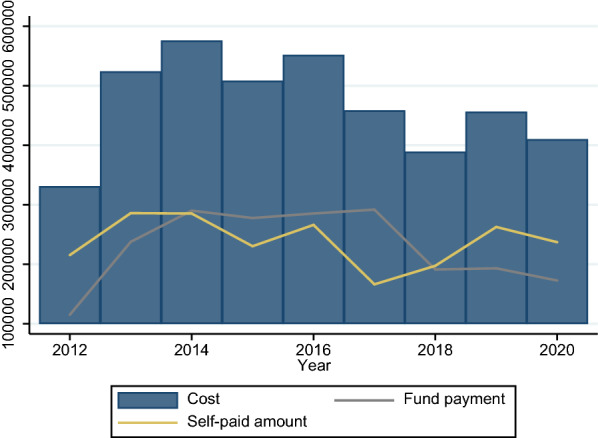
Cost of hemophilia patients

**Fig. 7:**
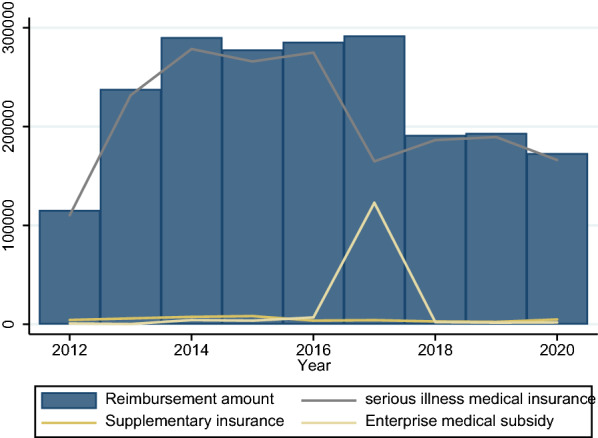
Cost reimbursement

Figure 6 reports the patient ‘s medical expenses and reimbursement. Figure 7 provides a more detailed report on reimbursement of medical insurance participating in reimbursement. In terms of medical expenses reimbursement for hemophilia patients, in general, the reimbursement rate is not high, around 50%. Among them, the serious illness medical insurance reimburses the vast majority of medical expenses, but its guarantee effect is not strong.

**Fig. 8:**
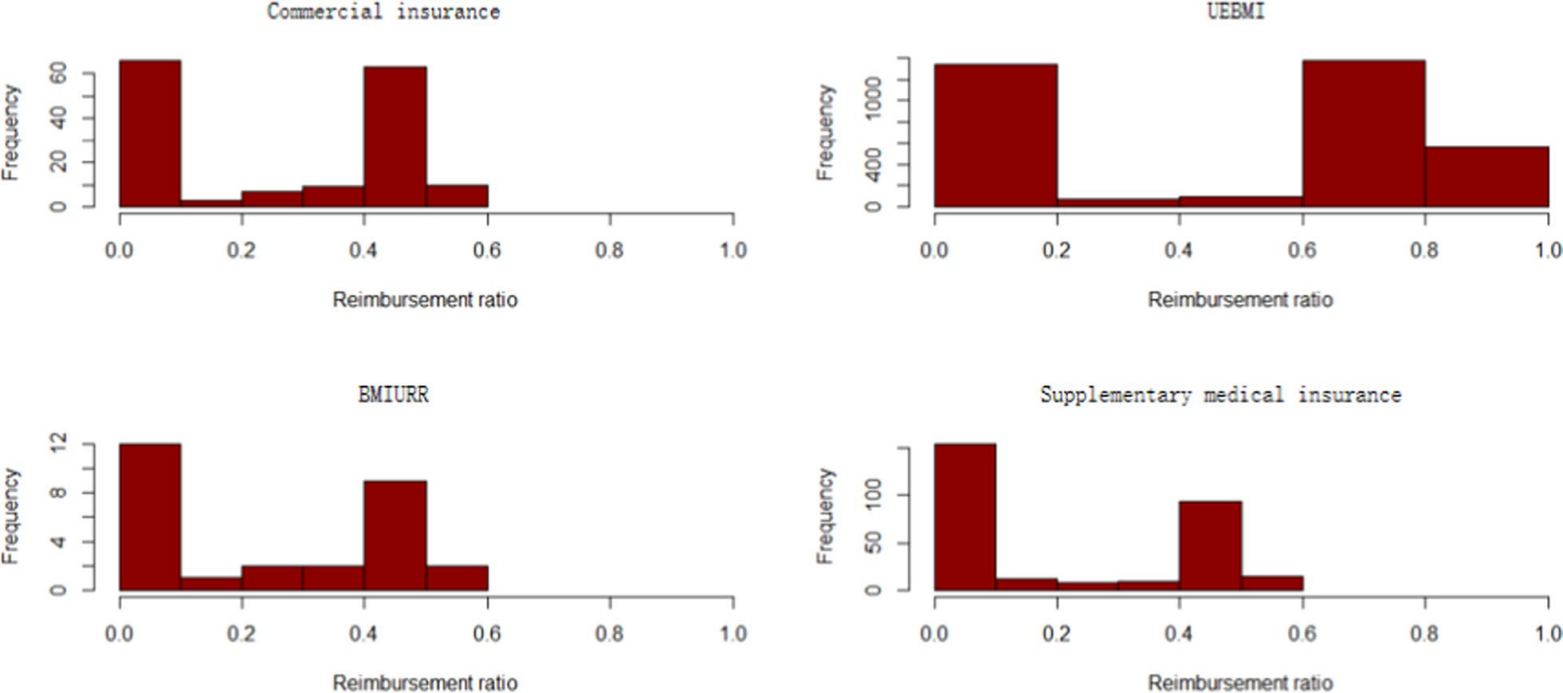
Expense reimbursement of Outpatient and emergency

**Fig. 9:**
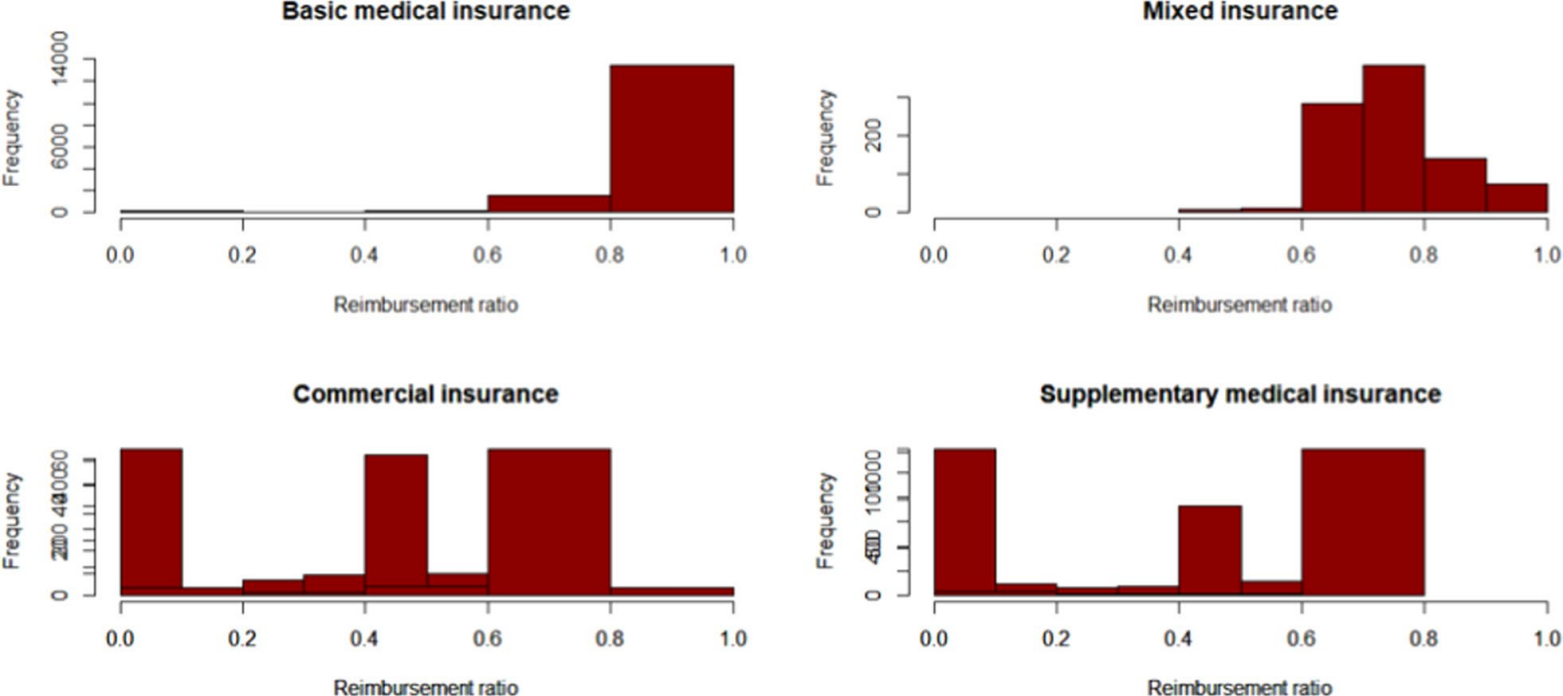
Expense reimbursement of outpatient special diseases

Figures 8 and 9 report the reimbursement ratio of different medical insurance for different patients. Reimbursement varies greatly among different types of insurance. In terms of outpatient and emergency costs, the reimbursement rate of medical insurance for urban employees is relatively high, which can reach more than 80%. The guarantee effect of insurance types is poor, and the reimbursement rate is all below 60%. At the same time, even with the same kind of insurance, the reimbursement ratio between different patients is also far away, the reimbursement rate of some patients can reach 98%, while the expenses of other patients can hardly be reimbursed. This is related to the current medical insurance system in China. Many rare disease drugs are not included in the medical insurance catalogue, and cannot be reimbursed naturally. Commercial insurance does not fully play a complementary role in social health insurance, resulting in significant differences between patients. Compared with outpatient and emergency expenses, the reimbursement ratio of special expenses is higher as a whole, and most patients can reach more than 60%. Similar to outpatient and emergency reimbursement, basic medical insurance has the strongest guarantee effect, followed by mixed insurance.

### Benchmark regression of medical service utilization affecting medical resource consumption

Firstly, the outpatient and emergency costs of hemophilia patients were analyzed by regression analysis. Table 2 shows the regression results of the total medical expenses. It can be seen that in terms of outpatient and emergency expenses, under the condition of controlling other factors unchanged, compared with patients without medical insurance, the consumption of medical resources in patients with medical insurance has a weak growth trend. The insured patients of basic medical insurance for urban employees will consume the most medical resources, followed by those of basic medical insurance for urban residents. Commercial insurance and supplementary medical insurance also promote the utilization of medical services for hemophilia patients. The type of medical insurance has a certain impact on the consumption of medical resources by hemophilia patients. The reasons for the above different results are that there are great differences in the scope and proportion of reimbursement among different medical insurance systems, resulting in different utilization of medical services for residents.

In the control variables, gender had a significant impact on the patient ‘s medical resource consumption. Because the incidence of hemophilia in men was significantly higher than that in women, the resource consumption of male patients was significantly higher than that of female patients. Age and the number of diseases also have a significant indigenous impact on the consumption of medical resources of patients. For each unit of increase in age, patients pay an average of 0.6 units less medical expenses.

**Table 2:** The estimation results of total outpatient and emergency medical expenses

Variables	Type				Sex	Age	Number of diseases
	UEBMI	BMIURR	Supplementary medical insurance	Commercial insurance			
Regression coefficient	670.3182	635.9383	564.762	598.4944	−304.7111	−0.5815	−91.5538
Robust standard error	0.172	0.218015	0.252	0.226916	< 0.001***	< 0.001***	< 0.001***
Sample size	4341	4341	4341	4341	4341	4341	4341

*** *p*< .001

Table 3 reports the regression results of western medicine cost, inspection cost, inspection cost and material cost. The regression results of medication costs are basically consistent with the results of total costs. The difference between the results of supplies costs, diagnosis costs, and treatment costs and the overall regression results may be due to the fact that these three costs are relatively small and the difference is not significant.

**Table 3:** Sub-item expenses estimation results

Variables	Type				Sex	Age	Number of diseases
	UEBMI	BMIURR	Supplementary medical insurance	Commercial insurance			
Medication cost	533.541 (0.277)	607.119 (0.239)	430.029 (0.383)	395.846 (0.424)	−343.004 (<0.001***)	−0.765 (<0.001***)	−76.759 (<0.001***)
Supplies cost	16.949 (0.484)	9.212 (0.718)	18.417 (0.450)	15.539 (0.525)	1.376 (0.667)	0.001 (0.901)	−0.352 (0.427)
Diagnosis cost	6.764 (0.879)	−0.602 (0.990)	−0.325 (0.994)	0.859 (0.985)	−6.303 (0.281)	−0.010 (0.524)	−1.290 (0.112)
Treatment cost	53.479 (0.634)	−2.041 (0.986)	62.369 (0.581)	48.018 (0.672)	8.845 (0.551)	0.019 (0.632)	−9.737 (<0.001***)
Sample size	4341	4341	4341	4341	4341	4341	4341

*** *p*< .001

Secondly, the outpatient special diseases costs of hemophilia patients were analyzed by regression analysis. Table 4 shows the regression results of the total medical expenses. It can be seen that the type of medical insurance has a significant indigenous impact on the consumption of medical resources of patients. When other factors remain unchanged, patients without medical insurance will consume the most medical resources, and there is no significant difference in the consumption of medical resources between patients with multiple mixed insurance and patients without medical insurance. Compared with the patients without medical insurance, the consumption of medical resources in patients with medical insurance is less. Commercial insurance has the strongest effect on the reduction of medical resources consumption, followed by supplementary medical insurance, and finally basic medical insurance for urban employees. Outpatient special diseases is mainly aimed at 22 special diseases. Compared with general outpatient, the cumulative medical expenses are relatively high. The reimbursement scope of medical insurance is limited and the reimbursement ratio is low. Therefore, medical insurance cannot promote the medical utilization behavior of patients.

In the control variables, gender has no significant indigenous effect on the consumption of medical resources of patients, and age and the number of diseases have significant indigenous effects on the consumption of medical resources of patients. Among them, for each additional unit of age, patients pay an average of 26.6 yuan less for medical expenses, which is inconsistent with our initial projections, perhaps due to more hemophilia than when they were young. This is not the focus of this article’s explanatory variables, so this article no longer does too much explanatory discourse. For each additional unit of the number of diseases, patients pay an average of 1574.3 yuan of medical expenses, consistent with the prediction of this article.

**Table 4:** The estimation results of total outpatient special diseases

Variables	Type				Sex	Age	Number of diseases
	UEBMI	Commercial insurance	Supplementary medical insurance	Mixed insurance			
regression coefficient	−1466.884	−3169.471	−1868.375	−343.834	−2117.899	−26.682	1574.259
robust standard error	< 0.001***	< 0.001***	< 0.001***	0.1575	0.0787	< 0.001***	< 0.001***
Sample size	18.401	18.401	18.401	18.401	18.401	18.401	18.401

*** *p*< .001

Table 5 reports the regression results of medication cost, treatment cost, diagnosis cost and supplies cost. The regression results of medication costs are basically consistent with the results of total costs. The difference between the results of treatment cost, diagnosis cost and supplies cost and the overall regression results may be due to the small amount of these three costs, and the difference is not obvious. The cost of diagnosis and treatment of the disease mainly includes the cost of surgery and nursing. The number of services is relatively fixed relative to the medication cost, and the influence of patients’ or doctors ' behavior is relatively small.

**Table 5:** Sub-item expenses estimation results

Variables	Type				Sex	Age	Number of diseases
	UEBMI	Commercial insurance	Supplementary medical insurance	Mixed insurance			
Medication cost	−1431.413 (<0.001***)	−3169.431 (<0.001***)	-1858.440 (<0.001***)	−400.692 (0.147)	−2352.905 (0.085)	−26.881 (<0.001***)	2152.586 (<0.001***)
Supplies cost	−1.949 (0.855)	−5.909 (0.646)	−13.643 (0.256)	−8.358 (0.497)	−3.052 (0.960)	−0.266 ( 0.001 **)	360.455 (<0.001***)
Diagnosis cost	12.526 ( 0.055)	13.434 (0.089)	13.998 (0.058)	16.127 (0.033*)	−14.765 ( 0.693)	0.139 (0.006 **)	46.112 (<0.001***)
Treatment cost	8.625 (0.469)	−12.022 (0.404)	35.665 (0.008 **)	24.669 (0.073)	142.859 (0.036 *)	−0.239 (0.010**)	−8.848 (0.287)
Sample size	18,401	18,401	18,401	18,401	18,401	18,401	18,401

* *p* < .05; ** *p* < .01; *** *p*< .001

## Discussion

Inequity in patient healthcare utilization can have a significant impact on patients’ healthcare resource consumption. With “Healthy China” becoming a keyword in this era, it is essential to achieve the equity of medical service utilization of patients with rare diseases to further promote social equity and justice. Based on the results, our study has important policy implications in the following three aspects.

Firstly, the medical security system should expand its coverage for patients with hemophilia. Our study has shown that some related disease drugs are not included in the medical insurance catalogue so they cannot be reimbursed. It leads to a huge difference in the reimbursement proportion between different patients under the same insurance type. According to relevant studies, this problem also exists for the coverage of the remaining rare diseases. Therefore, it is necessary to broaden the coverage of various medical insurance, and include more rare disease drugs in the medical insurance catalogue. We should provide corresponding support for patients with different types of diseases so as to better promote equity under the same type of insurance.

Secondly, the reimbursement level of medical expenses for patients with hemophilia should be appropriately improved, so as to effectively alleviate the economic burden of patients and their families. The study found that only outpatient large payment afford reimbursement for the majority of medical expenses, while other payments do not play effective roles in sharing the economic risks of patients. Similarly, the level of reimbursement of medical expenses for patients with the remaining types of rare diseases should be increased to reduce the risk of poverty caused by the disease and better contribute to the achievement of economic equity.

Finally, we should narrow the differences in the effects of different types of medical insurance security. The study found that in terms of reimbursement, the reimbursement ratio of UEBMI was significantly higher than that of other types of insurance. In terms of the consumption of medical resources, the effects UEBMI on the increase of medical resources consumption are also higher than that of other types of insurance. It reflects the differences in the security effects of different types of medical insurance.

## Conclusion

On conclusion, unequal utilization of medical services will have a significant impact on the consumption of medical resources of patients. The disparity in medical expenditure among different hemophilia patients is quite obvious, and the drug expense accounts for a large proportion of the total cost. The ratio of reimbursement is generally low, and there is a wide difference in the amount of reimbursement of different types of medical insurance. The resource consumption of patients with different medical insurance types differs a lot. During the process of constructing a better medical insurance system, we should pay special attention to the disadvantaged groups in the utilization of medical service resources, so as to improve the health condition and promote equity and justice for the whole society.

By analyzing the effect of type of health insurance on health care resource consumption among hemophiliacs, the study in this paper shows that differences in reimbursement rates between different types of health insurance will significantly affect the health care resource consumption of the insured population, which in turn leads to unequal health care resource consumption among different enrollment cohorts. That is, in addition to the urban-rural, regional, and individual income factors identified in the established literature, insurance participation status is also one of the possible causes of inequality in health care utilization. This study both expands the literature on inequality in health care resource consumption and fills the research gap regarding health care resource consumption in rare disease cohorts.

## Data Availability

The research data used to support the findings of this study are available from the corresponding author upon request.
